# Experiences of help-seeking from professional services for a child or young person’s mental health concerns during the pandemic: A qualitative study

**DOI:** 10.1371/journal.pone.0297417

**Published:** 2024-04-16

**Authors:** Frances Mathews, Simon Benham-Clarke, Tamsin J. Ford, Suzanne Hill, Katharine Sadler, Tamsin Newlove-Delgado

**Affiliations:** 1 University of Exeter Medical School, Exeter, United Kingdom; 2 Developmental Psychiatry, University of Cambridge, Cambridge, United Kingdom; 3 NatCen Social Research, London, United Kingdom; University of Ghana, GHANA

## Abstract

**Introduction:**

The immediate response to the Covid-19 pandemic saw school closures and a shift in provision to online health services for children and young people experiencing mental health concerns. This study provides mental health and referral services with an insight into difficulties experienced as well as recommendations on potential improvements.

**Methods:**

Semi-structured interviews with 11 parents and six young people. Reflexive thematic analysis was used to analyse the data.

**Results:**

Parents and young people reported mixed experiences on accessing mental health support. Priorities and pressures on health services impacted the likelihood of choosing to seek and being able to obtain help. Parents and young people had varying expectations and experiences in help-seeking during the pandemic which were also impacted by others’ experiences and views. For many, the relationship with the professional they were in contact with impacted their mental health treatment. Provision was sometimes accessed via private services due to long waiting lists or problems that did not “meet threshold”.

**Conclusion:**

Understanding the experiences of seeking mental healthcare during the pandemic can inform improvements to access to services at a time when people are most vulnerable. Accessible provision other than private services needs to be made for those on waiting lists. For those who do not meet service threshold, intermediary support needs to be secured to prevent unnecessary exacerbation of symptoms and prolonged problems. If schools are to remain the hub for children and young people’s mental health services, they should be considered essential services at all times.

## Introduction

Prompt access to assessment and suitable treatment for children and young people with mental health concerns are fundamental to improving outcomes [1, 2]. Prior to the Covid-19 pandemic, population surveys across high income countries reported that less than half of those meeting criteria for a psychiatric diagnosis were in contact with specialist mental health services [3, 4]. Commonly reported barriers to seeking professional help for mental health concerns [5] include a lack of information, embarrassment, and concerns around patient-therapist confidentiality. One Australian study found that a fifth of young people professionally assessed as having a disorder did not perceive a need for care, and that only half of those who did recognise a need reported mental health service use [6]. Once help is sought, other frequently cited factors likely to limit access to treatment include unclear referral criteria and high thresholds [7]. Research in the UK found that on average a quarter of children and young people referred into Child and Adolescent Mental Health Services (CAMHS) were assessed as not meeting the threshold for access, and those who did may be met with long waiting times for assessment and treatment [8–10].

Restrictions imposed during the Covid-19 pandemic, with school closures and limits to face-to-face contact outside the household, heightened concerns regarding help-seeking and access to treatment for mental health [11, 12]. In many countries, the focus on services switched to treat those affected by Covid-19. As a result, the availability and delivery of usual health care including mental health was severely disrupted [8, 13]. In England, following the initial lockdown in March 2020, child and adolescent mental health (CAMH) hospital admissions and referrals to specialist services via education and primary care fell by over 8% [8]. There was also a drop in primary care prescriptions of antidepressants and primary care presentations of self-harm, depression and anxiety among young people over a similar time period [14]. The impact of the pandemic on help-seeking behaviour was also evident in England’s Mental Health of Children and Young People (MHCYP) survey in 2021, where 26% of parents reported not seeking help for a child with a probable mental disorder between Summer 2020 and Spring 2021 due to the pandemic [15]. The impact on young people’s help-seeking behaviours appeared most marked, with more than half of those with a probable mental health disorder reporting they had not sought help. Changes in help-seeking behaviour were not limited to mental health. In one study of people with serious non-Covid-19 conditions, restrictions were perceived as limiting opportunities for symptom recognition, as well as leading to feelings of concern or fear about seeking help [16].

Despite this initial fall in help-seeking behaviour and mental health service contact, studies of population prevalence in England suggest an overall increase in probable mental health disorders among children and young people, with a rise in 5 to 16 year olds from one in nine in 2017 to one in six in 2020, which was sustained in 2021 and 2022 [3, 15, 17, 18]. An systematic review of the international impact of Covid-19 on children and young people’s mental health suggested an overall slight deterioration in broader measures of mental health, although findings were mixed [19].

This qualitative study aimed to complement existing routine and survey data by providing insight into why parent and young people chose to seek help from professional services during the pandemic for mental health concerns. To our knowledge, this is the first study to specifically explore experiences of mental health-related help seeking in this group during Covid-19. Understanding these experiences will paint a clearer picture for professionals and policy makers. We hope to inform service design and optimise outreach and targeted support. This is particularly important as current research indicates the most disadvantaged continue to be those least likely to seek support for their mental health needs [2, 3, 9, 20, 21].

## Methods

Ethical approval for this study was obtained for this study from University of Exeter College of Medicine and Health Research Ethics Committee and Cambridge Psychology Research Ethics Committee (PRE.2021.047). Written informed consent was obtained from all participants prior to participation as approved in ethics.

The original survey obtained ethical approval from West London and GTAC Research Ethics Committee. Data sharing was approved by National Health Services (NHS) Data Access Request Service (DARS) (DARS-NIC-331532-B5T0C). All methods were carried out in accordance with relevant guidelines and regulations.

### Participants and recruitment

The sample for this study was drawn from the Mental Health of Children and Young People in England (MHCYP) 2020 survey which invited all those who participated in MHCYP 2017. The initial survey comprised a population probability sample of children and young people aged 2 to 19 and their parents, drawn from the NHS Patient Register database [22]. These surveys were designed to provide England’s National Statistics on child mental health, with the 2020 follow-up also focussing on the impact of Covid-19. The MHCYP surveys collected data on a range of topics, with the main measure of mental health in the 2020 survey being the Strength and Difficulties Questionnaire (SDQ) [23], a brief questionnaire covering emotional and behavioural difficulties which can be used to identify probability of mental disorder [24]. Both the MHCYP 2017 and 2020 follow-up survey also included questions on help-seeking and contact with services with regards to their child’s (for parents) or their own (for young people aged 17 to 23) mental health. Participants from the MHCYP 2020 follow-up [18] who met eligibility criteria were invited to participate in this follow-on research study. The criteria were that the participants had consented to being contacted about further research, and that they had responded to the question, ‘in the past year have you or <Name> been in contact with any of these people because of worries about his/her emotions, behaviour, concentration or difficulties in getting along with people’? [3]. Participant data received were stored in the University of Exeter’s Secure Data Research Hub (SDRH).

### Procedure

228 parents of children aged 5–16 and 201 young people aged 17–22 were contacted by letter or email with information about the study. All participants were asked to read, complete and return the study consent form before their interview commenced, and received a gift voucher as a token of appreciation for their time. The developed topic guide was piloted with young people and parents who participated in our patient and public involvement groups, and in interviews, with suggested amendments incorporated into the final guide. Semi-structured interviews (see S1 Appendix Topic Guide) were based on participant’s experiences of contact with services with concerns about their child’s (as reported by the parent) or their own (as reported by the young people) mental health during the Covid-19 pandemic. Interviews were conducted by telephone or online at a convenient time for the participant.

The research team conducted a total of 17 interviews: 11 with parents of children aged 6–16 years, slight majority female; and six with young people aged between 17–22 years, majority female. Participants were broadly spread across English regions, with fewer parents from the South of England and fewer young people from the North of England.

### Analysis

We selected reflexive thematic analysis approach to explore these data as the six phase process reflected the overall research question of understanding experiences of help-seeking during the pandemic [25, 26].

Interviews were recorded and transcribed using an approved transcription service. Data were managed using NVIVO 1.6.1 [27]. All researchers recognised that their role as researchers and their experiences of healthcare services may influence their expectations and interpretations of the data, and this was explicitly discussed as part of the analysis process.

**Fig 1:**
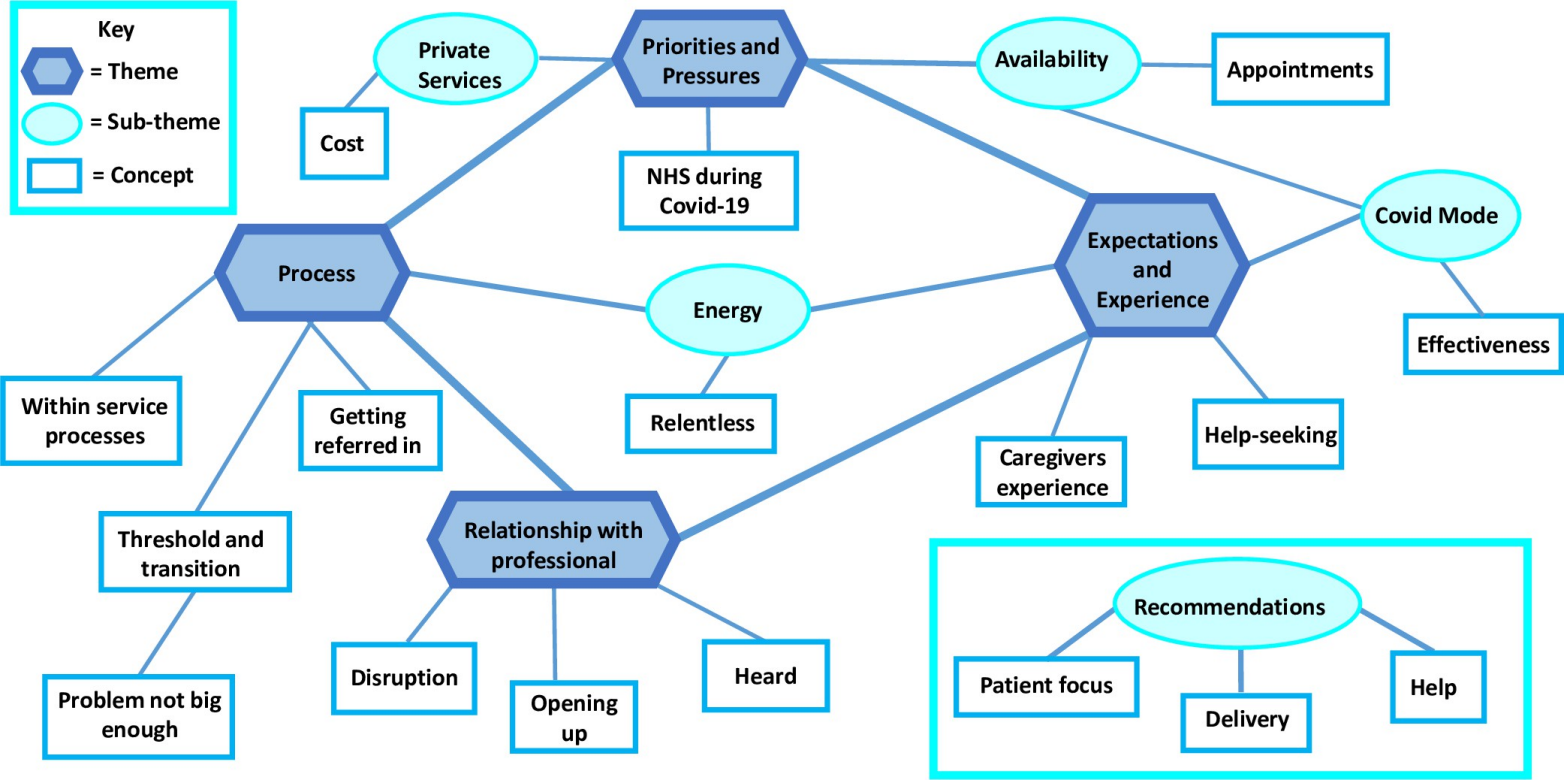
Mind map showing themes, sub-themes and concepts derived from participant interviews.

To explore the range of meanings within the data, an inductive method towards the data analysis was taken. The research team (FM, TND and SBC) each read 2 of the transcripts as part of initial familiarisation with the data. These data were approached flexibly and openly to generate initial concepts. These 6 transcripts were then double coded by one other member of the team (FM, TND and SBC). Initial codes were discussed at length, with all codes noted. The remaining transcripts were coded by a single researcher (FM) who generated additional codes where identified. Additional codes were then discussed and further refined with the research team, to develop a final coding structure (FM, TND & SBC). Candidate themes were then developed and defined by the research team (FM & TND). Each theme was reviewed to consider a) how well the theme was supported by the underlying data, b) how distinct the themes were from another, and c) how they related to one another (see Fig 1). The decision was made to retain sub-themes which describe key and distinct aspects within the overarching theme. A more deductive approach was used to code responses to questions about to parents and young people’s recommendations to improve experiences of professional service access given their opinions were specifically solicited (see S1 Appendix Topic guide). This was an isolated direct question fell outside the remit of the other open questions and the researchers (FM & TND) took the decision to analyse and present the data in a separate section. We used a simple analysis of grouping responses into categories which lay under the then pre-defined theme, Recommendations.

Following agreement of the generated themes, anonymous quotes of the transcripts were selected to support the concepts. The final themes were then reviewed and agreed by the full research team, again considering internal homogeneity and external heterogeneity (FM, TND and SBC). The research team (FM and TND) approached the full research team (KS, SBC, SH & TF) to review, refine and agree the final themes, being receptive to and incorporating feedback on the structure and linkage of the themes, sub-themes and codes.

## Results

Four themes were generated (see Fig 1) relating to the experiences of help-seeking for mental health concerns during the pandemic which included: Process; Priorities and pressures; Expectations and Experiences; and Relationship with Professional.

### Theme: Process

Participants described in detail the processes they went through in order to access support during socially restricted periods during lockdown.

#### Getting referred in

During the pandemic, participants most commonly reported seeking referral via their General Practitioner (GP), with many reporting the referral process to be long. One parent reported relief at receiving support:

[P1] “I feel quite fortunate, really. I know that some people’s experiences haven’t been like that. But I felt that the GP service was very… they really did listen and didn’t dismiss what I was saying to them when I first called, and they were very thorough”

Participants reported how lockdown had delayed access to services, with one parent noting how this affected their child’s experience:

[P2] “she had what you call an initial assessment in the January/February and then, of course, the pandemic started… ..So they weren’t seeing people… .And then I think she started to be seen in the following September, so about nine/ten months later, and she was just about to start college…… And it was very difficult for [young person], because she really wouldn’t talk to people about her problems”

Referrals could also work well, with those in need receiving help or treatment quickly despite lockdown constraints:

[P1] “which would have been a month, 6 weeks, I would say, from my first initial appointment with the GP, she was seen by a [Service] clinician”

#### Within service processes

Problems were also reported in the process of moving from one service location to another, which, in one instance, created a long delay in patient information being transferred. Other difficulties were also recalled, with one young person who told of the process of liaising with professionals as they juggled communication attempts alongside daily life:

[YP1] “Like, they’d ring you randomly, and when it’s no caller ID, you can’t then just ring them back, because that’s what it would be, so you can’t just ring them back straightaway. And then I feel like, at the time, I was quite anxious already with college and balancing everything, and then that just kind of added to it a little bit more.”

Difficulties in accessing help within services were also raised, with one parent reporting the impact of specialist child services on their child’s care:

[P3] “… and I know that people were busy and stretched and probably for her it seemed like everything else she had going on, she was perhaps at the end of her tether, I don’t know, but that’s where I feel really that clinic for children ideally would have still been running, and there would have been a specialist nurse who would have dealt with it better.”

#### Threshold and transition

Both parents and young people discussed how hard it was when the problem was seen as being ‘not big enough’ by professionals and services. One parent reported being discouraged by their spouse from help-seeking as the spouse’s professional opinion was that the problems were ‘manageable’. One reported being told by professionals that they would not receive service support as the child’s behaviours were not exhibited when receiving only online help:

[P4] “We were in that time when we couldn’t do things. Over virtually… I just think [son] is very good at putting a show on so when he would engage and look at the screen, he very much said and showed what he thought those people wanted to hear. And then when he was on the bed bouncing rolling screaming at me afterwards, they didn’t see any of that, whereas if it was face-to-face they might have seen more of that.”

Some participants believed there was a mismatch in how they felt about the problems experienced and what they were supposed to be able to deal with, and for how long given no one was certain how long the pandemic would last:

[P5] “And four months is a terrible long time for a kid that doesn’t really want to live anymore–and she was telling me quite frequently that she didn’t see the point in living, it’s too hard”

One parent voiced alarm at how extreme their child’s difficulties would need to be in order to warrant service support:

[P2] “she said she has to just go on this waiting list, it’s very long and she may not meet the threshold. Goodness knows what threshold you need to be on to get help”

Reaching the age restriction for services also disrupted access to care. For example, after being on a waiting list for two years after pandemic related backlog, one parent was informed that their child would be moved onto a waiting list for adult services as opposed to receiving immediate short-term help when a space for support arose:

[P5] “And then in the end they said, ‘well, because [name]’s turning 18 at Christmas, really it’s only four months away, there’s nothing really else we can do. Just, when she turns 18, refer to adult services’.”

#### Energy

Many participants experienced the process of getting help as demanding a large amount of energy. One parent who was actively urged to complain about the early discharge of the child recalled the efforts required in making a complaint:

[P5] “they said that the only way you’ll feel better is if you make a complaint and that we’re urging all parents to make complaints. But it takes a lot of energy to make a complaint.”

One young person reported it was difficult for them to call the surgery owing to their depression, and that was made more difficult when their GP left:

[YP2] “Because it was a bit hard to get a doctor in the pandemic as well”

Some parents reported that pandemic meant they had to work harder in getting help for their child, even taking on the role of services, for example managing the transition to another service by orienting the young person which needed to be done in person. Securing support via the telephone was found to be particularly lengthy, which required a ‘relentless’ approach:

[P6] “you ring them and you get the whole feature of Covid and if you’ve got symptoms call this number, go here, go there, do this, do that and then you finally get to the options of being able to press whatever to get through to whoever you need to and then half the time you just get cut off straightaway so you’ve got to keep ringing back again and again and again”

### Theme: Priorities and pressures

#### NHS priorities during Covid-19

Many parents and young people thought that the additional pressures on services that they experienced were due to Covid-19. They talked about the impact that Covid-19 had on ‘usual care’, appearing to accept that staff numbers were low due to sickness or leaving, with those who remained being under pressure more than usual. There was also a perception that Covid-19 was the main priority for the NHS. According to one young person:

[YP3] “(the) NHS was just focusing on Covid cases and putting everything else on the backburner.”

Participants felt this message was being corroborated by medical professionals within the health service:

[P1] “certainly from what I’ve heard through clinicians saying, as well, is that they’ve had to take on a lot more patients since the pandemic than they were before, and their services have been stretched quite a lot”

One parent reported part of the decision not to access NHS based help was having feedback from family with professional knowledge of the healthcare system:

[P7] “I don’t think [name] felt that it was 100% necessary. [name] feels there’s always someone who’s worse off than you.

Parents in particular reported feeling grateful for any support that they were given:

[P1] “from that point of view, I’m even more grateful that we’ve been able to get into the service fairly quickly”

Some young people and parents expressed that it was understandable that services were not running as usual:

[P8] “Listen, generally, I think the support that’s available is phenomenal and I just think that it’s been overstretched and it’s difficult because of the situation of not being able to see people face-to-face”

Related to this, some parents reported not seeking help, because they did not want to increase the burden from Covid-19:

[P3] “as parents we perhaps weren’t as willing to try and get help because of the pandemic, we were trying to deal with it on our own rather than make work for people, I suppose, put more pressure on the health service”

#### Availability

Participants had varying experiences of service availability during Covid-19. Some found they were able to get referrals and face-to face appointments quickly, the transition to online support was effective (see Covid Mode) and they were rapidly supported in accessing help:

[P1] “there is a helpline for the service, so if I have an immediate concern, they can arrange to get hold of them and call me back straightway.”

However, particularly following the first lockdown, availability of any services was described as being particularly low:

[P7] “We did look at sort of privately and had got someone recommended. But then when you went on, with Covid, it was either not face to face or they were only dealing with adults and not children, at that point.”

#### Private services

In response to long waiting lists some parents sought more immediate support from private services. One parent reported that their GP actively signposted them towards private services:

[P2] “so she (GP) was really helpful. I think she called up people that she knew and she came back to me with a name and we got in touch with that lady and luckily she had a slot that was free and she started to speak to [name].”

Some parents described relief at having been able to obtain and afford a private therapist:

[P2] “In the meantime, I received a letter from the… I think it’s called CA-… CAMHS… .… ..I received a letter from them saying that they wouldn’t be able to offer any help for [name], that she didn’t meet the threshold [laughs], so I was really relieved that we’d at least managed, and fortunate that we could afford to get her help”

Both parents and young people reported the financial strain of paying for professional mental health help:

[YP4] “I think the only issue was it was quite expensive. I had a grandparent who was happy to pay that for me. If that hadn’t have been the case then I guess it would have been a different story”

### Theme: Experiences and expectations

#### Help-seeking

Positive experiences and expectations of help-seeking were recounted, often with one impacting the other. One parent found that support during the pandemic was easier to access than people had told them it would be:

[P1] “Well, I feel quite fortunate, really. I know that some people’s experiences haven’t been like that. But I felt that the GP service was very… they really did listen and didn’t dismiss what I was saying to them”

Others reported how hard it was to access support. A young person considered the messaging around seeking support for mental health concerns which did not seem to match the provision:

[YP1] “They have posters around, loads of posters that were saying like, “Seek help if you feel like this, and such and such.” And then obviously I’ve spoken with my teachers quite directly, told them, and told them about specific aspects of it… . It didn’t really seem like it got referred on to anybody else above, because there was obviously like their own mental health officer in college, but it didn’t seem to really go beyond anything that I was telling other people”

Some young people felt that what was offered did not meet their expectations. One young person needed more guidance:

[YP1] “Yeah, I just wanted more… rather than people asking me like, “What do you want us to do?” I wanted them to be like, “This is what you should do, or this is what you can do.” At that time, when you are younger, and you are looking towards to your teachers, and you are still in that mind-set where you want them to tell you”

#### Covid mode

The change of ‘mode’ from face-to-face to online during lockdown received mixed reviews. One young person accepted the new mode as they wanted to move on with treatments rather than wait:

[YP5] “It wasn’t ideal, obviously in person would have been better but understanding what you couldn’t do at that time, I was happy with it because I’d rather just finish it rather than say “We can put it on hold and wait until who knows when Covid’s going to be done,”

However, others felt that telephone and online support meant that their child’s problems were unable to be properly assessed. With telephone contact, children were said to “parrot” or “mask” responses as the therapist could not ‘see’ their body language. One parent told of how distressing being on an online video call was for their child given the child’s difficulties:

[P9] “Well for [name] it went terribly…[name] doesn’t like having her picture taken, she doesn’t like being on camera, she doesn’t like her reflection. She was very distressed, she broke down, it just didn’t work for her, it was bad. It wasn’t the way to do it. The screen obviously creates obstacles”

Others found that professional flexibility reduced the pressure of attending appointments which were far away:

[P1]”I think, initially, they suggested that we needed to go to [Town] every fortnight to get that done but, again, with the travelling distance, we’ve managed to negotiate with our local GP to take the physical checks and email them to the [Service]”

#### Caregiver’s experience

Some parents reported how the child’s mental health difficulties had impacted their own lives. One parent had to give up their job whilst others sourced couples counselling. Parents called for more support to be available during these times:

[P1]”I felt as though I needed to talk to them about my work and how I balanced my work with the needs of [Name]”

They also reported feeling exhausted by chasing services for help, feeling worried and having to ‘walk on eggshells’ around the child whilst waiting for help.

Many parents reported feeling frustrated as the experience with the service did not meet the level of professionalism they had expected. One main concern raised by parents was the lack of contact and liaison between them and the professional leaving them unsure about how to best support their child:

[P10] “she’s obviously creating a confidential situation with [Name], so he is free to say whatever he wants, which I totally understand. But as a parent that is quite challenging because you also want to know what you can do to help.”

### Theme: Relationship with professional

Most parents and young people described the type of relationship they had between themselves and the professional and how it impacted their experience. Parents also talked about how valuable it was to have a professional who listened to them and signposted to alternative sources of help when they themselves could not assist any further:

[P2] “I guess because she was more understanding and she knew she couldn’t help by getting us referred to anybody. But she called me in her own time on my mobile and gave me the referral because I’d asked her, “Please help me, I just don’t know where to begin””

Parents and young people also reported difficulties, with some feeling like their concerns were not taken seriously:

[P11] “[clinician] said, ‘lots of kids say they’re going to kill themselves, it’s not like they’re going to do it and if she was really serious, she would have done it by now.”

They also reported worries about being able to share the problems with the professional. This was considered especially hard for those for whom it was hard to open up to a family member. One young person expressed conflicting feelings:

[YP5] “I don’t know if I should just give it a chance because maybe it was because it was Covid times when it was lockdown so she was busy, or whether it’s just her as a person and maybe we don’t work well together.”

In some cases, relationships with professionals were disrupted or ended due to staff sickness:

[P5] “‘it sounds like she’s not going to be back anytime soon, can you not reallocate [name] somebody?’ In the end, they reallocated her… .… .… ..she had six sessions with her and then she went off sick with stress as well”

### Recommendations

Parents and young people were asked for any suggestions they had to improve the help-seeking process given their own experiences. These recommendations are tabulated (see Table 1) to make them accessible, and to reflect focussed responses to a specific question in the topic guide (see Methods & S1 Appendix).

**Table 1:** Recommendations from parents and young people on improving access to help for mental health.

Area	Recommendations	Supporting Quotes from Parents (P) and Young People (YP)
Patient focused improvements	Clearer communication about how to get help Being followed-up Provide earlier access enabling earlier assessment ‘same person’ point of access Clinicians/therapists to have more of an upfront and honest approach with young people Information / support on where to seek help when the child or young person doesn’t fit the current care model A tailored approach to the child’s needs and symptoms	[YP1] “I wanted them to be like, “This is what you should do, or this is what you can do.” [P5] “instead of leaving people that are on the waiting list for a year…. . .I think to make people know that you’ve not forgotten them” [P10] “if we’d have had that help earlier, we might have gone to the assessment process earlier” [YP6] “doctors should probably have a designated person to speak about, like a designated GP to speak about these things” [YP1] “when you are talking to teenagers, like they know when you are sidestepping around something and they know when you are not being straightforward, and I think they just want more honesty, I suppose, in what you are saying towards them” [P4] “my biggest challenge along this process is that [child] self-harms significantly and that is not recognised in a child so young” [P5] “I don’t think there can be a one bill that fits all system.”
Mode / type of support	Need for flexibility with method of therapy delivered as on-line support were not always found to be suitable or effective	[P9] “to have it in person, not in zoom, with the screen. [name] doesn’t want to see [them]self
Information and help	Young people wanted more services to be available Parents wanted more information on the type of mental health disorders. and support for the child’s mental health concerns including better signposting Parents wanted clearer information about when their child was likely to be seen by services / how long they would be waiting for help	[YP2] “Try and make themselves [medical professionals] a bit more available” [P5] “I want to put a booklet together for parents, just to show what there is out there in [county] and what each one means and I just cannot understand why there isn’t something like that” [P5] “the communication over the phone, linking in with families and kids more, if there is a pandemic, just letting them feel like they’re not forgotten”

## Discussion

This study is to our knowledge the first to examine parents’ and young peoples’ experiences of help-seeking from services in England for mental health concerns during Covid-19. Our findings reflect a variety of experiences of the process of seeking and receiving help during a period of unusual and restrictive practices, rapid change and uncertainty. We identified four themes: Process; Priorities and Pressures; Expectations and Experiences; and Relationship with Professional. This study also highlights Recommendations from parents and young people and wider implications for policymakers and professionals.

Our results show the *process* of help-seeking experienced by parents and young people was varied, and common challenges emerged. Many reported feelings of frustration and distress owing to long waiting lists, which continue to affect child mental health services [8]. Long waits for treatment are found to exacerbate symptoms and impact development [2, 9, 10]. For others, the referral process was unclear and left them feeling uncertain whether they met the threshold for service access. Thresholds and access criteria have been widely cited as a significant barrier to service access, with a recent report finding that a third of children and young people who are referred into services did not meet the threshold [9]. Unclear processes around referral combined with a high threshold for service access are likely to increase distress, especially when a long wait for services is unsuccessful [7]. Clarity around the process of help-seeking is particularly important for young people who are much less likely to seek professional help for mental health concerns, and who may also be transitioning from child to adult services [3, 6]. Our study also highlights the potential mismatches between messaging and advertising which encourage young people to reach out for support, and what is available in practice, which risks further discouraging help-seeking [8, 9]. Accounts of help-seeking via education settings were reported less than expected, given that education professionals have previously been shown to be a main point of contact and referral for children needing support [3, 28]. One explanation for this may be changes in education provision during the pandemic, as much contact both for parents and children with schools and early-years settings shifted online, reducing opportunities to identify or discuss concerns [29].

Our findings suggest that perceptions of the *prioritisation and pressures* of Covid-19 directly influenced decisions made by some young people and parents not to seek professional help, which echoes a Canadian study by Markoulakis et al. [10]. They reported that young people and caregivers had ‘negative perceptions’ of service availability during Covid, and uncertainties over what was on offer. This is in addition to the usual barriers perceived and experienced by young people in accessing professional based mental health support [30, 31]. While those experiencing other socio-economic challenges experienced more marked barriers to service access others have raised concerns about differential impacts of the pandemic on the most vulnerable population groups [2, 3, 9, 21]. In our study, some participants also appeared acutely aware of the pressures being felt by health professionals, and seemed to wish to avoid adding to these. Several commented on changes in the professionals supporting them due to staff sickness, which contributed to their perceptions of a system under strain. The challenges faced by health care services during this period were openly disclosed by professionals who reported struggling to provide the quality of care they felt patients should receive [32, 33]. Expectations of services seemed to be directly influenced by these perceptions. In our interviews, a number of participants were keen to express gratitude for any help that was received, or acknowledged when telling their own story that others were worse off. Some also attributed problems or shortcomings in their care to the pressures of Covid. A ‘sense of duty’, and concerns over burdening healthcare were also reported by Parretti et al. [16] in their study of help-seeking for serious non-Covid health concerns in the UK. In Parretti’s study, some participants explicitly connected such feelings to public messaging and media reporting during the pandemic, but this was less obviously evident in the accounts of our participants. The experiences we report may contribute to the initial drop in referrals reported by many studies using routine data [33, 34]. The combination of decisions made not to access help and the fluctuation in levels of help available are of particular concern alongside reports of sustained increase in probable mental health disorders in England’s national child mental health follow-up surveys [15, 17, 18]. Not only that, the recent spike in referrals by 81% compared to the same period in 2019 suggests a backlog of suppressed demand [34]. England is not alone in this pattern of service drop and demand, as mental health care professionals in other countries report referrals exceeding pre-pandemic rates [33].

Parent and young people’s *experiences and expectations* of mental health services during Covid-19 were also affected by the rapid move to online-based support revealing both positive and negative aspects. Some parents raised concerns about the accuracy and depth of on-line assessments. In one case, the experience of being on camera was acutely distressing to the child. This is also discussed in Markoulakis et al’s [10] study of caregiver’s experiences of youth mental health during the pandemic where caregivers reported difficulties in engagement and rapport online, voicing a preference for in-person care. Research with professionals echoes some of these concerns, with mental health staff finding that whilst online consultations had many advantages, it was often not accessible or suitable for those considered most vulnerable including those experiencing domestic abuse, family conflict, or those where the therapeutic relationship was more difficult to establish [20]. Unsuitability of on-line support for children was also a concern raised in Werling et al. [33] who highlighted the need for play and interaction in the provision of effective mental health care for children which was difficult to achieve online. For some participants in our study, the continuation of access to support was of greater importance and seemed to outweigh the delivery of online therapy.

These experiences linked to the role of *relationship with professionals*, another of our themes which is well established as important to healthcare [5, 32, 35]. Those who had a positive relationship with the mental health professional typically described hallmark traits experienced in any good relationship including trust, flexibility and respect [5]. Conversely, some participants described experiencing the behaviour of professionals as undermining or unacceptable, which added to the stress of the situation and resulted in delays in access to care or disengagement with treatment. Improving access to mental health care for children and young people must be a priority for pandemic recovery. Services were already under pressure prior to Covid, but even a slight deterioration in mental health on a population level can have a much greater impact on population level service provision [17, 19]. Importantly, our participants’ recommendations of how to improve their experience centred around early access, availability, communication and clearer information (see Table 1). These recommendations can be used alongside existing research outlined in this study to inform the mental health and wellbeing plan, now to be incorporated within the NHS Long term plan [36]. Recent reports show just over half of all clinical commissioning groups (CCGs) in England spend the targeted 1% of their budget on child and young people’s mental health services. The recent spike in demand for treatment and already long waiting lists means an increase in service provision is desperately needed as the current backlog of need is being faced by an already stretched service [8, 9, 17, 19, 33]. In addition, prevention and targeted intervention for those at greater risk and less likely to access support are also needed to stem the flow.

The delivery of access and availability of support presents a significant challenge and perhaps one of the most alarming realisations from Covid-19 restrictions was the inability for children and young people to access mental health school-based hubs. In March 2021, in response to ‘levelling-up’, the Department of Health and Social Care (DoHSC) pledged some of the £79 billion for children and young people’s mental health to be used to improve school based mental health support [37]. The continuous delivery of these hubs needs serious consideration in the event of future school closure ensuring the most vulnerable receive the support they need. It is also fundamental to understand the limitations of virtual service provision. Both parents and young people recommended increased accessible mental health information, appropriate/tailored sign posting and available service support. Where a child or young person is waiting for support or a service reject a referral, clear signposting to other services which have not already been exhausted by families is required [38].

### Strengths and weaknesses

This qualitative study is as far as we know the first to explore parent and young people’s experiences of help-seeking for mental health concerns during the pandemic in England. The study participants were drawn from a representative population study in England, but as a relatively small sample may not reflect the full range of experiences. Not all those participating in 2017 or 2020 agreed to contact, and although not formally analysed, it seems likely that those facing greater adversity and poorer mental health would be less likely to participate [39]. We are aware that young people are increasingly more likely to seek informal sources of support for mental health concerns than parents of children, with this paper focusing solely on formal support [3, 15, 18]. Finally, interviews took place up to 2 years after the initial lockdown in March 2020. We are therefore reliant on recall of parents and young people about their experiences.

## Conclusion

Our research suggests that restrictions arising from the Covid-19 pandemic made the process of help-seeking more complex, and perception of pressure on services may have affected decisions to seek help. The quality of the relationship with the mental health professional shaped the experience and expectations of those getting help. Accurate planning and implementation of accessible support for those waiting for assessment or treatment seems essential in light of the current demand.

## Supporting information

S1 Checklist Human participants research checklist. (DOCX)

S1 Appendix Topic guide. (PDF)

## List of abbreviations

MHCYP: Mental Health of Children and Young People
CCE: Children’s Commissioner for England
CAMH: Child and Adolescent Mental Health
SDRH: Secure Data Research Hub
