# Supplementary material for: Experiences of help-seeking from professional services for a child or young person’s mental health concerns during the pandemic: A qualitative study
Source: PLoS One. 2024 Apr 16;19(4):e0297417. doi: 10.1371/journal.pone.0297417 (PMC11020827; doi:10.1371/journal.pone.0297417)
Supplement: S1 Appendix — (PDF) [file pone.0297417.s002.pdf]

SERVICE CONTACT INTERVIEW: Parent version

HELP SEEKING DURING THE PANDEMIC

We want to ask about seeking or receiving help for mental health concerns about your child **during the pandemic:** (from the beginning of the pandemic **when the UK first went into lockdown March 2020 until the end of September 2021** (last 18 months)

Semi-structured interview: Experiences of services during the Covid-19 pandemic

Q1) We're interested in hearing more about your experiences of services and seeking help during the pandemic – can you tell us about these in a bit more detail?

What sort of support or help were you looking for?

Who did you get in touch with/have contact with? Did you get in contact or did someone else?

What happened when you contacted the service?

Use these prompts:

- Did you/your child see anyone?
- Who did you see?
- Was this online/telephone/in person?

What did they do?

- Was your child given a diagnosis
- Was your child prescribed drugs for these problems
- Did they have any talking therapies?
- Were you referred anywhere else?
- If so, where

How did you find the process of getting help?

- How easy or difficult was it?
- What went well?
- What did not go well?/what problems were there?

Would you say that the treatment/support/service did what you were hoping for?

- Why/why not?
- How did this affect your child?
- How did it affect you and your family?

Looking back on your experience with (trying to get help/getting help)

- Was there anything that could have been done differently to help?
- Are there any changes that services could make to help children, young people and families in case there is a future lockdown or future pandemic?

Q2)
What is the current situation now?(e.g. on waiting list, have another appointment booked, discharged, recovered?) *open question*
